# LSMMD-MA: scaling multimodal data integration for single-cell genomics data analysis

**DOI:** 10.1093/bioinformatics/btad420

**Published:** 2023-07-08

**Authors:** Laetitia Meng-Papaxanthos, Ran Zhang, Gang Li, Marco Cuturi, William Stafford Noble, Jean-Philippe Vert

**Affiliations:** Google Research, Brain Team, Google, Brandschenkestrasse 110, Zurich 8002, Switzerland; Department of Genome Sciences, University of Washington, 3720 15th Ave NE, Seattle, WA 98195, United States; eScience Institute, University of Washington, 3910 15th Ave NE, Seattle, WA 98195, United States; Department of Genome Sciences, University of Washington, 3720 15th Ave NE, Seattle, WA 98195, United States; eScience Institute, University of Washington, 3910 15th Ave NE, Seattle, WA 98195, United States; Google Research, Brain Team, Google, 8 Rue de Londres, Paris 75009, France; Apple ML Research, Apple, 7 Av. d’Iéna, Paris 75116, France; Department of Genome Sciences, University of Washington, 3720 15th Ave NE, Seattle, WA 98195, United States; Paul G. Allen School of Computer Science and Engineering, University of Washington, 185 E Stevens Way NE, Seattle, WA 98195, United States; Google Research, Brain Team, Google, 8 Rue de Londres, Paris 75009, France; Owkin, Inc., 14/16 Bd Poissonnière, Paris 75009, France

## Abstract

**Motivation:**

Modality matching in single-cell omics data analysis—i.e. matching cells across datasets collected using different types of genomic assays—has become an important problem, because unifying perspectives across different technologies holds the promise of yielding biological and clinical discoveries. However, single-cell dataset sizes can now reach hundreds of thousands to millions of cells, which remain out of reach for most multimodal computational methods.

**Results:**

We propose LSMMD-MA, a large-scale Python implementation of the MMD-MA method for multimodal data integration. In LSMMD-MA, we reformulate the MMD-MA optimization problem using linear algebra and solve it with KeOps, a CUDA framework for symbolic matrix computation in Python. We show that LSMMD-MA scales to a million cells in each modality, two orders of magnitude greater than existing implementations.

**Availability and implementation:**

LSMMD-MA is freely available at https://github.com/google-research/large_scale_mmdma and archived at https://doi.org/10.5281/zenodo.8076311.

## 1 Introduction

Modality matching in single-cell genomics data analysis can enhance our understanding of the relationships between cellular modalities and help us resolve cell states. In this problem, single-cell measurements collected using two or more different types of assays are projected into a shared space or are otherwise matched across modalities, with the goal of achieving insights into the joint multimodal dataset. Most existing multimodal models rely on learning cell representations in each modality in a joint low-dimensional space (Welch *et al.* 2019, Cao *et al.* 2020, Jin *et al.* 2020, Stark *et al.* 2020, Gayoso *et al.* 2021, Hao *et al.* 2021, Raimundo *et al.* 2021, Cao and Gao 2022). MMD-MA (Liu *et al.* 2019, Singh *et al.* 2020) is one such method that has shown promising results on datasets containing several thousand cells in each modality. However, thanks to new single-cell technologies, the size of single-cell datasets has increased significantly in the past 2 years, now reaching several hundreds of thousands to millions of cells (Papatheodorou *et al.* 2020, Hao *et al.* 2021, Rozenblatt-Rosen *et al.* 2021). These datasets cannot be analysed by current implementations of MMD-MA due to memory issues.

More precisely, MMD-MA (Liu *et al.* 2019) is a multimodal approach that maps each cell in each modality to a shared, low-dimensional representation space. The linear mappings from the input spaces to the representation space are learned by minimizing an objective function composed of several terms: (i) a “matching” term based on the squared maximum mean discrepancy (MMD) with a Gaussian radial basis function (RBF) kernel to ensure that the different modalities overlap in the representation space, (ii) two “noncollapsing” penalties to prevent trivial solutions, and (iii) two “distortion” penalties to ensure that as much information from the input data as possible is captured in the shared representation. More details about the method are provided Supplementary Appendix Section A. However, current implementations of MMD-MA (Liu *et al.* 2019, Singh *et al.* 2020) scale quadratically as a function of the number of cells in memory and runtime, which is prohibitive for datasets with more than a few thousand samples (see Supplementary Table SA4).

To increase the scalibility of MMD-MA, we introduce LSMMD-MA, a reformulation and PyTorch implementation of MMD-MA that overcomes the memory explosion issue. To achieve this, we (i) reformulate MMD-MA’s optimization problem in the primal, which is beneficial when the number of cells is larger than the number of informative features and (ii) implement the MMD matching term with the CUDA-based KeOps library for symbolic matrices (Charlier *et al.* 2021), tailored to handle matrices that do not fit in RAM or GPU memory. The resulting algorithm scales only linearly in memory with the number of cells and can handle up to a million cells in each modality.

## 2 Materials and methods

### 2.1 Reformulating MMD-MA in the primal

Let $X\in R^{n_{x}\times p_{x}}$ (respectively, $Y\in R^{n_{y}\times p_{y}}$) be the data matrix of the first (respectively, second) modality, where $n_{x}$ (respectively, $n_{y}$) is the number of cells in the first (respectively, second) modality and $p_{x}$ (respectively, $p_{y}$) is the number of features in the first (respectively, second) modality. The goal of MMD-MA is to learn two mappings from the input spaces $R^{n_{x}}$ and $R^{n_{y}}$ to a shared representation space $R^{d}$. We focus specifically on linear mappings, as in the original publications (Liu *et al.* 2019, Singh *et al.* 2020), where mappings are parameterized with dual variables $\alpha_{x}\in R^{n_{x}\times d}$ and $\alpha_{y}\in R^{n_{y}\times d}$ such that the embedding of the first (respectively, second) modality is $XX^{⊤}\alpha_{x}$ (respectively, $YY^{⊤}\alpha_{y}$). Instead, we equivalently parameterize the mappings by primal variables $W_{x}\in R^{p_{x}\times d}$ and $W_{y}\in R^{p_{y}\times d}$, such that the embedding of the first (respectively, second) modality is $XW_{x}$ (respectively, $YW_{y}$). We can then rewrite the MMD-MA optimization problem in the primal: where $\lambda_{1}$ and $\lambda_{2}$ are hyperparameters. Under the assumption that n≫p≫d for each modality, efficiently implementing the primal loss (Equation 1) scales better than implementing the dual loss, as shown in Supplementary Table A2. The primal loss does not require the computation and storage of the linear kernel matrices $XX^{⊤}$ and $YY^{⊤}$, which are O($n^{2}$) in time and memory, and the penalty and distortion terms are not O($n^{2}$) in runtime anymore. However, computing the MMD term remains O($n^{2}$) in runtime and memory if we implement it naively. A description of MMD-MA in the dual and a comparison between the formulations of MMD-MA and LSMMD-MA are available in Supplementary Appendix Sections A and B.


(1) $$\begin{array}{c} \underset{W_{x},W_{y}}{\min}L_{\text{primal}}(W_{x},W_{y})=\underset{W_{x},W_{y}}{\min}[\mathrm{MMD}{\left(XW_{x},YW_{y}\right)}^{2}\\ +\lambda_{1}(\text{pen}(W_{x})+\text{pen}(W_{y}))+\lambda_{2}(\text{dis}(X,W_{x})+\text{dis}(Y,W_{y}))], \end{array}$$

### 2.2 Using KeOps

To overcome the O($n^{2}$) memory burden of computing the MMD term, we implement it using the CUDA-based Map-Reduce scheme of KeOps (Charlier *et al.* 2021). This allows us to compute the MMD term without instantiating the n×n Gaussian RBF kernel in memory, using symbolic matrix computation with O(*n*) memory complexity, as detailed in Supplementary Appendix Section D. KeOps therefore optimizes (Equation 1) with a linear memory complexity and also improves runtime by a significant multiplicative factor when the number of samples is >1000.

### 2.3 Implementation

We make four algorithms available, including LS-MMDMA and three variants: primal formulation without KeOps, dual formulation with KeOps, and dual formulation without KeOps (an efficient implementation of the original algorithm). The code is implemented in PyTorch (Paszke *et al.* 2019) and can run on CPU or GPU. The package is open source with an Apache license, available at github.com/google-research/large_scale_mmdma. It is referenced on PyPI and can be installed with the command: pip install lsmmdma. Details about I/O, command line instructions and tutorials are given in the Readme.md and in the examples folder.

## 3 Results and conclusion

We tested the scalability of the implementation of LSMMD-MA (primal formulation with KeOps) against three comparison partners (primal formulation without KeOps, dual formulations with KeOps and dual formulation without KeOps) and against the two original implementations (Liu *et al.* 2019, Singh *et al.* 2020) which focus on the dual formulation in TensorFlow (Abadi *et al.* 2016) and PyTorch (Paszke *et al.* 2019), respectively. Additionally, Singh *et al.* (2020) proposes to use the linear time approximation of MMD for large numbers of samples (>5000) (see Lemma 14 in Gretton *et al.* 2012).

**Figure 1.**
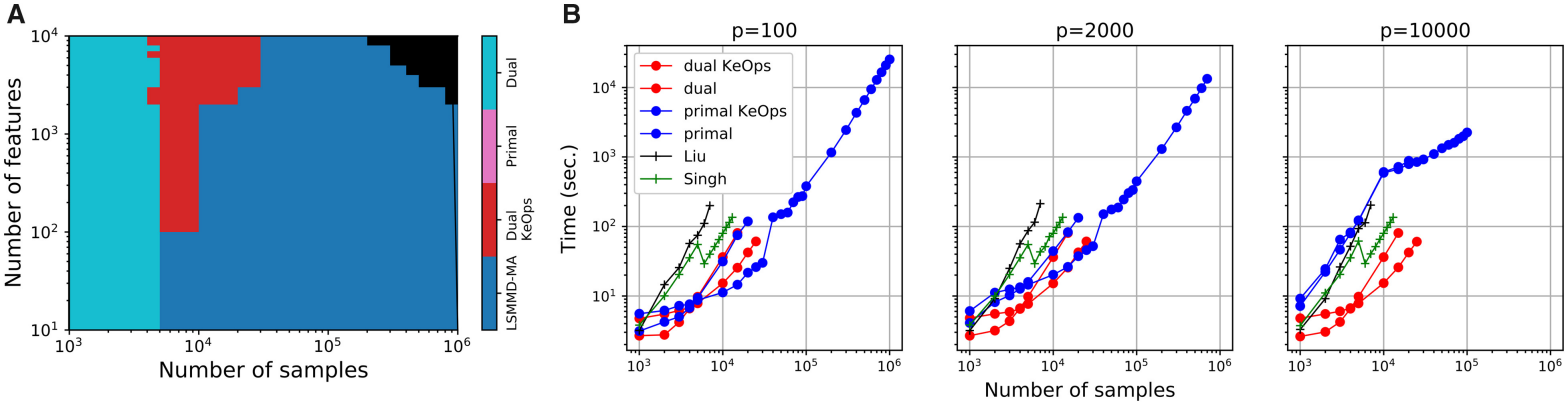
(A) Fastest MMD-MA variant as a function of the number of samples and the number of features. The black region in the top right corner means that all variants ran out of memory. (B) Runtime as a function of number of cells for different implementations of MMD-MA, when the dimension *p* of the input data varies. The black and green dotted lines with cross markers correspond to the original implementations of MMD-MA as written by  Liu *et al.* (2019) (black) and Singh *et al.* (2020) (green). The runtime for different values of *p*, from 100 to 10 000, is shown in Supplementary Appendix Fig. A1.

We ran all algorithms on simulated datasets of different sizes where the latent space is shaped as a branch (see lsmmdma/data/data_pipeline.py in GitHub and Supplementary Appendix Section G for more details). All algorithms were run for 500 epochs and a low-dimensional representation of dimension d=10. A V100 GPU (16GB) was used for the experiments. We observe that LSMMD-MA, using the primal formulation and KeOps, scales to one million cells in each modality, whereas the original implementations runs out of memory for >14 000 cells (Fig. 1). In the same figure, we can also notice that our dual implementations are faster than the original implementations irrespective of the number of samples. We also show that LSMMD-MA obtains good accuracies, as measured by the Fraction Of Samples Closer than The True Match (FOSCTTM) (Liu *et al.* 2019), on a selection of the simulated datasets (see Supplementary Appendix Section E). MMD-MA and LSMMD-MA have the same optimal objective values as LSMMD-MA is a reformulation of MMD-MA. Furthermore, we show that both algorithms obtain similar FOSCTTM performance on 12 synthetic datasets with varying numbers of features and samples (see Supplementary Appendix Section B).

As a proof of principle, we also ran LSMMD-MA on a real-world CITE-seq dataset containing 90 261 human bone marrow mononuclear cells, with 13 953 gene IDs for the gene expression modality and 134 proteins for the protein marker modality (Luecken *et al.* 2021). We obtain an FOSCTTM of 0.22 after 100 000 epochs (10.3 h) with LSMMD-MA, which would have been infeasible with previous versions of MMD-MA. More details about the preprocessing of the dataset and the hyperparameters are available in Supplementary Appendix Section G. We additionally compared LSMMD-MA with baselines such as Procrustes superimposition (with and without aligned data) (Gower 1975), LIGER (PyLiger) (Liu *et al.* 2020, Lu and Welch 2022) and Harmonic alignment (Stanley *et al.* 2020) and show that LSMMD-MA is competitive (see Supplementary Appendix Section H for detail).

These results suggest that an optimized implementation, exploiting the primal formulation and taking advantage of the KeOps library, are key to building a multimodal model that scales to the size of current single-cell datasets.

## Supplementary Material

btad420_Supplementary_Data

## Data Availability

The simulations used in this article are available in GitHub at https://github.com/google-research/large_scale_mmdma and the CITE-seq dataset is available from NCBI GEO under accession GSE194122.
