# Supplementary material for: LSMMD-MA: scaling multimodal data integration for single-cell genomics data analysis
Source: Bioinformatics. 2023 Jul 8;39(7):btad420. doi: 10.1093/bioinformatics/btad420 (PMC10336029; doi:10.1093/bioinformatics/btad420)
Supplement: btad420_Supplementary_Data [file btad420_supplementary_data.pdf]

LSMMD-MA: Scaling multimodal data integration for single-cell genomics data analysis

Appendix

Appendix

Notations used in the appendix

Let $X \in \mathbb{R}^{n_x \times p_x}$ be the data matrix of the first modality and $Y \in \mathbb{R}^{n_y \times p_y}$ the one of the second modality. n_x (resp. n_y) is the number of cells in the first (resp. second) modality and p_x (resp. p_y) is the number of features in the first (resp. second) modality. We denote by $K_x = XX^\top \in \mathbb{R}^{n_x \times n_x}$ and $K_y = YY^\top \in \mathbb{R}^{n_y \times n_y}$ the linear kernel matrices corresponding to both datasets.

A Description of `MMD-MA` with the dual formulation

`MMD-MA` [1] is a method for analyzing multimodal data that relies on mapping the observed cell samples to embeddings, using functions belonging to a reproducing kernel Hilbert space (RKHS). The authors build linear kernels for both domains and learn the coefficients of the embedding functions in the RKHS dual representation. In practice, the `MMD-MA` loss is composed of a) the squared MMD, a *matching* term, b) two *non-collapsing* penalties and c) two *distortion* penalties, one for each modality.

Using the notations described in the previous section, the loss of `MMD-MA` can be written as follows:

$$\min_{\alpha_x,\alpha_y} L_{dual}(\alpha_x, \alpha_y) = \min_{\alpha_x,\alpha_y} [\text{MMD}(K_x\alpha_x, K_y\alpha_y)^2 \\ + \lambda_1(\text{pen}(K_x, \alpha_x) + \text{pen}(K_y, \alpha_y)) + \lambda_2(\text{dis}(K_x, \alpha_x) + \text{dis}(K_y, \alpha_y))]$$

where $\alpha_x \in \mathbb{R}^{n_x \times d}$ and $\alpha_y \in \mathbb{R}^{n_y \times d}$ are the learned coefficients of the RKHS functions used to build the embeddings $K_x\alpha_x$ and $K_y\alpha_y$ of dimension d, λ_1 and λ_2 are two hyperparameters weighing the non-collapsing penalty ($pen(K, \alpha) = ||\alpha^T K\alpha - \mathbf{I}_d||_2$) and the distortion penalty, ensuring that the geometries in the low- and high-dimensional representations are comparable ($dis(K, \alpha) = ||K - K^T\alpha^T\alpha K||_2$). The RBF kernel is used to calculate MMD, introducing non-linearity in the matching term. One downside of the dual formulation is that the linear and gaussian kernel matrices require memory and runtime that scale quadratically as a function of the number of cells in terms of memory and runtime, which is prohibitive for large datasets.

B Primal (`LSMMD-MA`) and dual (`MMD-MA`) formulations side-by-side

In the dual, the mappings between original and latent spaces are parameterized with the dual variables $\alpha_x \in \mathbb{R}^{n_x \times d}$ and $\alpha_y \in \mathbb{R}^{n_y \times d}$, such that the embedding of the first (resp. second) modality is $XX^\top\alpha_x$ (resp. $YY^\top\alpha_y$). Instead, in the primal, we equivalently parameterize the mappings by primal variables $W_x \in \mathbb{R}^{p_x \times d}$ and $W_y \in \mathbb{R}^{p_y \times d}$, such that the embedding of the first (resp. second) modality is XW_x (resp. YW_y). The relationship between the two formulations can be given by $W_x = X^\top\alpha_x$ and $W_y = Y^\top\alpha_y$. Given this relationship, we can rewrite all the terms of `MMD-MA` in the primal as a function of (X, Y, W_x, W_y) instead of $(K_x, K_y, \alpha_x, \alpha_y)$:

- MMD term: $MMD(XW_x, YW_y)^2 = MMD(K_x\alpha_x, K_y\alpha_y)^2$
- penalty term: $||\alpha^\top K\alpha - \mathbf{I}_d||_2 = ||W^\top W||_2$
- distortion term: $||K - K^\top\alpha^\top\alpha K||_2 = ||XX^\top - XWW^\top X^\top||_2$

We observe that `LSMMD-MA` and `MMD-MA` obtain similar FOSCTTM scores on 12 synthetic datasets. We considered only datasets with a maximum number of samples of $10,000$ because `MMD-MA` runs out of memory for more than $14,000$ samples (see Table A1). For this comparison, we chose the multi-start experimental setting presented in [1], where the algorithm is run for several seeds and the best seed is chosen according to the smallest loss in the training set. We simulated our own synthetic dataset with a branch-like latent space with the `generate_data` function in lsmmdma/data/data_pipeline.py. We fixed the number of seeds to 15. For both algorithms, we stopped training at $50,000$ epochs. We observed that the losses do not decrease significantly anymore at this point. The scaling parameter of the Gaussian RBF kernel in the MMD term was set to 30, and the regularisation parameters were fixed $(\lambda_1, \lambda_2) = (\frac{0.01}{\sqrt{p}}, \frac{0.0001}{n\sqrt{p}})$, where n and p are the numbers of samples and features in each modality. We set the learning rate to $5.0 * 10^{-4}$ for `LSMMD-MA` and

to $1.0 * 10^{-6}$ for MMD-MA. Initialisation of the parameters was uniform in both cases, from 0 to 1 for LSMMD-MA and 0 to 0.1 for MMD-MA as in [2] and [1]. We add amsgrad=True to the optimizer of MMD-MA as suggested in [2]. We found that in practice this set of hyperparameters, selected according to [1], works well for a variety of datasets; however, most results are robust to changes of one to two orders of magnitude of the hyperparameters.

	$n = 1000$	$n = 5000$	$n = 10000$
$p = 100$	0.0001 vs. 0.0002	0.0002 vs. 0.0007	0.0015 vs. 0.0011
$p = 1000$	0.0031 vs. 0.0004	0.0009 vs. 0.001	0.0031 vs. 0.0033
$p = 2000$	0.0002 vs. 0.0003	8e-05 vs. 0.0006	0.0009 vs. 0.0015
$p = 6000$	5e-06 vs. 0.0002	0.0002 vs. 0.0006	6e-05 vs. 0.0005

Table A1: Comparison of FOSCTTM between LSMMD-MA (left) and MMD-MA (right). MMD-MA ran out of memory for a number of samples larger than 14,000 (see Appendix Section E for further results for LSMMD-MA).

C Runtime and memory requirements of LSMMD-MA and MMD-MA

Model	MMD-MA dual				MMD-MA primal			
	input	MMD	pen.	dis.	input	MMD	pen.	dis.
Runtime	$O(\mathbf{n^2}p)$	$O(\mathbf{n^2}d)$	$O(\mathbf{n^2}d + nd^2)$	$O(\mathbf{n^2}d)$	-	$O(\mathbf{n^2}d + pnd)$	$O(d^2p + d^2)$	if $n > p$: $O(p^2n + p^3 + p^2d)$ else: $O(n^2p + nd))$
Memory	$O(\mathbf{n^2})$	$O(\mathbf{n^2})$	$O(nd + d^2)$	$O(nd)$	$O(np + pd)$	$O(\mathbf{n^2} + nd)$	$O(d^2)$	$O(min(p, n) * (p + n + d))$

Table A2: Runtime and memory requirements for MMD-MA in the primal and dual forms without using KeOps. n is the number of cells, p the number of features and d the dimension of the embeddings. We remove the subscripts x and y for readability but the O notations of the table hold for each modality. Typically, $n >> p >> d$ for each modality.

We compute the distortion term in two different ways, depending on the number of samples and features. If the number of samples is smaller than the number of features ($n < p$), we compute the distortion as: $dis = ||XX^\top - XWW^\top X^\top||_2$. If the number of samples is larger than the number of features ($n > p$), we want to avoid computing $XX^\top$ which scales quadratically in n, in terms of memory and runtime. We therefore take advantage of the relationship between the trace of a matrix and the Frobenius norm and compute instead: $dis = \sqrt{Tr((I - WW^\top)X^\top X(I - WW^\top)X^\top X)}$.

D Brief introduction to KeOps and its Map-Reduce computations

KeOps [3] is a package that allows to compute efficiently, on GPUs, operations of the form a) calculation of a vector valued function based on input vectors (in our case, the Gaussian kernel) and b) reduction operation on a given axis (in our case, the average of the Gaussian RBF kernel).

The idea is based on a block by block map reduced scheme: the data is loaded from the device to the GPU memory only block by block. Once a block is loaded, the computation of the vector values function is done on the GPU, and the result of the reduction operation is stored in a running buffer. As a consequence, it is possible to have access to fast computations in the GPU without needing to load the entire matrix at once.

In our case, the bottleneck comes from the calculation of the average of the Gaussian RBF kernel in the MMD term. For example, for one million samples, the kernel matrix would be one million by one million which does not fit in GPU memory. The block-by-block scheme of

KeOps enables one to calculate the average of the Gaussian kernel without ever needing to instantiate the entire matrix. Detailed explanations and tutorials are available at: https://www.kernel-operations.io/keops/index.html

E FOSCTTM obtained with `LSMMD-MA`

The Fraction Of Samples Closer than The True Match (FOSCTTM) is a common metric to measure accuracy in modality matching. The lower the metrics, the better the algorithm. We tested `LSMMD-MA` on several synthetic datasets, as described in Appendix G. The experimental settings follow the ones described in the simulations of [1] and [2]. In both cases, the algorithm is ran several times (i.e. for several seeds) and the best seed is chosen according to the smallest loss in the training set (see [1]) or to the smallest FOSCTTM in the validation set (see [2]). We stopped training at $20,000$ epochs. The learning rate was fixed to 0.0005, the scaling parameter of the Gaussian RBF kernel in the MMD term was fixed to 4σ where σ was the average of the distance matrix, the regularisation parameters (λ_1, λ_2) were either $(\frac{0.1}{\sqrt{p}}, \frac{0.01}{n\sqrt{p}})$ or $(\frac{0.01}{\sqrt{p}}, \frac{0.0001}{n\sqrt{p}})$, where n and p are the number of samples and features in each modality. We show that `LSMMD-MA` can reach a very good performance for both experimental settings (Tables A5 and A4), as measured by FOSCTTM.

	$n = 1000$	$n = 10^4$	$n = 10^5$	$n = 5 * 10^5$	$n = 10^6$
$p = 100$	0.00173	0.00347	0.00337	0.00018	0.00011
$p = 2000$	0.0002	0.00025	0.00042	0.00003	-

Table A3: FOSCTTM obtained when selecting the smallest loss in the training set at the 20000th epoch. Similar results were obtained when selecting the best epoch based on the smallest loss.

	$n = 1000$	$n = 10^4$	$n = 10^5$	$n = 5 * 10^5$	$n = 10^6$
$p = 100$	0.00006	0.00012	0.00011	0.0001	0.00004
$p = 2000$	0.00014	0.00015	0.00007	0.00002	-

Table A4: FOSCTTM obtained when selecting the best FOSCTTM in the validation set at the 20000th epoch. Similar results were obtained when selecting the best epoch based on the smallest loss.

F Benchmark

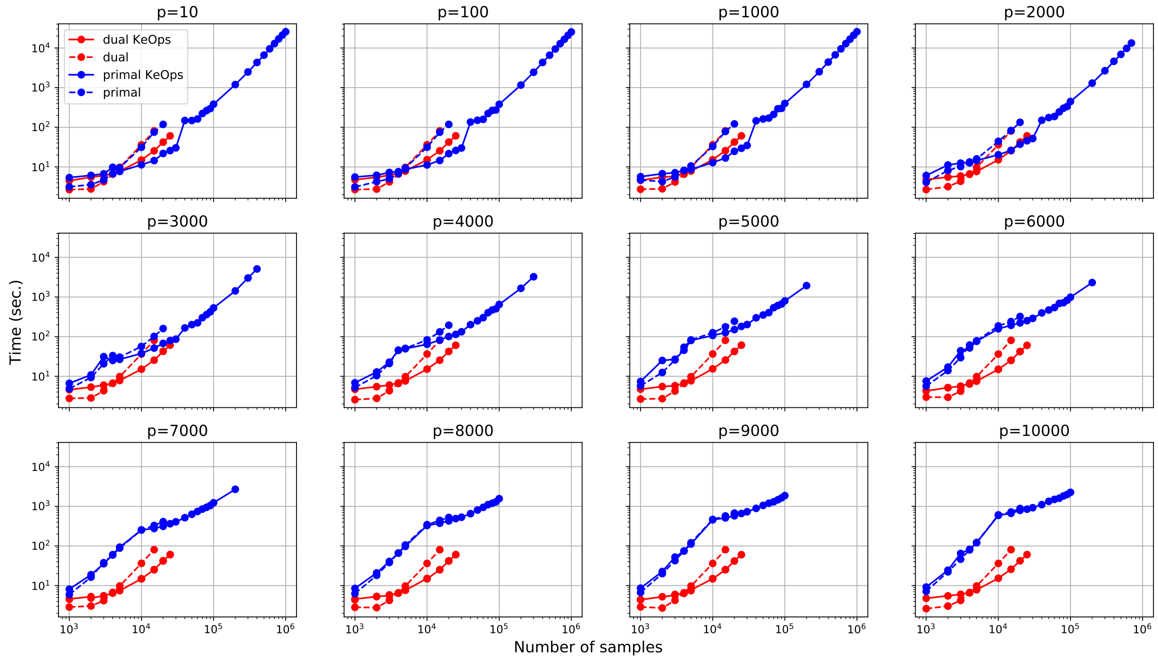

Figure A1: Runtime as a function of number of cells for different implementations of `MMD-MA`, when the dimension p of the input data varies. The algorithms ran for 500 epochs and a low-dimensional representation of dimension d = 10, on a V100 GPU (16GB).

G Datasets

The synthetic datasets used for the runtime experiments have a varying number of samples from 10^3 to 10^6 and a varying number of features from 10 to 10^4. To generate them, we used the `generate_data` function in lsmmdma/data/data_pipeline.py, with the `random_seed` argument fixed to 4 and the `simulation` argument set to 'branch'. The simulation process works as follows. A manifold (of shape 'branch') is generated in two dimensions. The resulting set of points is standardised. The points are then mapped to `p_feature` dimensions using a (2 x `p_feature`) mapping, sampled from a standard Gaussian distribution, resulting in a (`n_sample` x `p_feature`) matrix. Gaussian noise is then added to each element of the matrix. This simulation was first described in [1] and the latent space shaped as a branch aims at mimicking a development process for example.

The real-world dataset on which the last experiment is ran comes from [4], where datasets were made publicly available for the Neurips Competition Multimodal Single-Cell Data Integration. The dataset was already preprocessed to remove low quality cells and doublets. The counts were log-transformed. We selected the genes in the gene expression modality and ADT protein measurements based on a common gene ID, which resulted in 36 features for each modality. We used the same hyperparameters as in the simulations (see Section E in the Appendix) and ran `LSMMD-MA` for 100,000 epochs.

H Comparison to baselines

In this section, we compare LSMMD-MA to several widely used single-cell data integration methods.

Among potential comparison partners we note that

- `SCOT` [5] and `UnionCOM` [6] do not scale to a large number of cells ($n > 50,000$ cells). Indeed, `SCOT`'s runtime scales in $O(n^3)$, which is prohibitive on a CPU and scales in $O(n^2)$ in terms of memory which is prohibitive on a GPU. `UnionCOM` is a deep learning-based method that scales quadratically in terms of the number of samples memory-wise, which is prohibitive on a GPU.

- `SCIM` [7] is a deep-learning based approach that requires weak cell-label supervision, which is also not the setting we are tackling.

- Finally, several other approaches (matrix factorisation based or deep learning based), such as `totalVI` [8], `scMVAE` [9], `MOFA+` [10] and `Seurat v4` [11], require cell alignment which is not needed with LSMMD-MA.

We therefore compared LSMMD-MA to three baselines that can scale to a similar number of cells and are appropriate for the current setting. For our evaluation, we used the CITE-seq dataset and the simulation dataset described in Appendix Section B, with 10000 samples and 1000 features. The three methods are as follows:

- LIGER [12], in particular its Python version PyLiger [13], which is NMF-based. We used the provided GitHub code and adapted it to make it work with a more recent Python version. The simulated data was made positive by substracting the (negative) minimum value of each modality to the entire matrix. The real data was preprocessed according to the package guidelines. We ran PyLiger and varied the following parameters: the embedding dimension ($[10, 20, 30]$) and the lambda value ($[1, 5$ (default)$, 8, 10]$) which balances the importance of shared features compared to modality-specific ones.

- Harmonic alignment [14]. We note that using the GitHub code with the default parameters does not scale to large matrices, as it requires computing all the eigenvalues of an n by n matrix. We therefore reduced the number of eigenvectors used when decomposing the matrix. The real data and simulated data were preprocessed as for `LSMMD-MA`. We ran the algorithm varying the following parameters: the number of eigenvectors ($[10, 20, 40, 100]$) and the number of filters ([4 (default), 10].

- Procrustes [15]. We used Procrustes superimposition (`scipy.spatial.procrustes`) on the aligned modalities as a positive control and on the unaligned modalities to have a comparable setting to LSMMD-MA. The real data and simulated data were preprocessed as for `LSMMD-MA`.

We obtained the following results with the FOSCTTM metric:

models	LSMMD-MA	Procrustes (aligned)	Procrustes (unaligned)	LIGER (best)	Harmonic align. (best)
simulated data	0.003	0.003	0.50	5e-5	0.49
CITE-seq	0.22	0.23	0.49	0.49	0.48

Table A5: Comparison to baselines (FOSCTTM).

We observe that LSMMD-MA's performance remains comparable to the baselines.

References

[1] Liu, J., Huang, Y., Singh, R., Vert, J.P., and Noble, W.S. (2019). Jointly embedding multiple single-cell omics measurements. In 19th International Workshop on Algorithms in Bioinformatics (WABI 2019), *Leibniz International Proceedings in Informatics (LIPIcs)*, volume 143. pp. 10:1–10:13. doi:10.4230/LIPIcs.WABI.2019.10.

[2] Singh, R., Demetci, P., Bonora, G., Ramani, V., Lee, C., Fang, H., Duan, Z., Deng, X., Shendure, J., Disteche, C., et al. (2020). Unsupervised manifold alignment for single-cell multi-omics data. In Proceedings of the 11th ACM International Conference on Bioinformatics, Computational Biology and Health Informatics. pp. 1–10. doi:10.1101/2020.06.13.149195.

[3] Charlier, B., Feydy, J., Glaunès, J.A., Collin, F.D., and Durif, G. (2021). Kernel operations on the gpu, with autodiff, without memory overflows. Journal of Machine Learning Research *22*, 1–6.

[4] Luecken, M.D., Burkhardt, D.B., Cannoodt, R., Lance, C., Agrawal, A., Aliee, H., Chen, A.T., Deconinck, L., Detweiler, A.M., Granados, A.A., et al. (2021). A sandbox for prediction and integration of DNA, RNA, and proteins in single cells. In Proceedings of the Neural Information Processing Systems Track on Datasets and Benchmarks, volume 1, J. Vanschoren and S. Yeung, eds. (Curran).

[5] Demetci, P., Santorella, R., Sandstede, B., Noble, W.S., and Singh, R. (2022). Scot: Single-cell multi-omics alignment with optimal transport. Journal of Computational Biology *29*, 3–18.

[6] Cao, K., Bai, X., Hong, Y., and Wan, L. (2020). Unsupervised topological alignment for single-cell multi-omics integration. Bioinformatics *36*, i48–i56.

[7] Stark, S.G., Ficek, J., Locatello, F., Bonilla, X., Chevrier, S., Singer, F., Rätsch, G., and Lehmann, K.V. (2020). Scim: universal single-cell matching with unpaired feature sets. Bioinformatics *36*, i919–i927.

[8] Gayoso, A., Steier, Z., Lopez, R., Regier, J., Nazor, K.L., Streets, A., and Yosef, N. (2021). Joint probabilistic modeling of single-cell multi-omic data with totalVI. Nature Methods *18*, 272–282. doi:10.1038/s41592-020-01050-x.

[9] Zuo, C. and Chen, L. (2021). Deep-joint-learning analysis model of single cell transcriptome and open chromatin accessibility data. Briefings in Bioinformatics *22*, bbaa287.

[10] Argelaguet, R., Arnol, D., Bredikhin, D., Deloro, Y., Velten, B., Marioni, J.C., and Stegle, O. (2020). Mofa+: a statistical framework for comprehensive integration of multi-modal single-cell data. Genome biology *21*, 1–17.

[11] Argelaguet, R., Cuomo, A.S., Stegle, O., and Marioni, J.C. (2021). Computational principles and challenges in single-cell data integration. Nature biotechnology *39*, 1202–1215.

[12] Liu, J., Gao, C., Sodicoff, J., Kozareva, V., Macosko, E.Z., and Welch, J.D. (2020). Jointly defining cell types from multiple single-cell datasets using liger. Nature protocols *15*, 3632–3662.

[13] Lu, L. and Welch, J.D. (2022). Pyliger: scalable single-cell multi-omic data integration in python. Bioinformatics *38*, 2946–2948.

[14] Stanley III, J.S., Gigante, S., Wolf, G., and Krishnaswamy, S. (2020). Harmonic alignment. In Proceedings of the 2020 SIAM International Conference on Data Mining (SIAM), pp. 316–324.

[15] Gower, J.C. (1975). Generalized procrustes analysis. Psychometrika *40*, 33–51.
